# Similarity Measure Learning in Closed-Form Solution for Image Classification

**DOI:** 10.1155/2014/747105

**Published:** 2014-06-26

**Authors:** Jing Chen, Yuan Yan Tang, C. L. Philip Chen, Bin Fang, Zhaowei Shang, Yuewei Lin

**Affiliations:** ^1^Faculty of Science and Technology, University of Macau, Taipa 999078, Macau; ^2^Chongqing University, Chongqing 400030, China; ^3^University of South Carolina, Columbia, SC 29208, USA

## Abstract

Adopting a measure is essential in many multimedia applications. Recently, distance learning is becoming an active research problem. In fact, the distance is the natural measure for dissimilarity. Generally, a pairwise relationship between two objects in learning tasks includes two aspects: similarity and dissimilarity. The similarity measure provides different information for pairwise relationships. However, similarity learning has been paid less attention in learning problems. In this work, firstly, we propose a general framework for similarity measure learning (SML). Additionally, we define a generalized type of correlation as a similarity measure. By a set of parameters, generalized correlation provides flexibility for learning tasks. Based on this similarity measure, we present a specific algorithm under the SML framework, called correlation similarity measure learning (CSML), to learn a parameterized similarity measure over input space. A nonlinear extension version of CSML, kernel CSML, is also proposed. Particularly, we give a closed-form solution avoiding iterative search for a local optimal solution in the high-dimensional space as the previous work did. Finally, classification experiments have been performed on face databases and a handwritten digits database to demonstrate the efficiency and reliability of CSML and KCSML.

## 1. Introduction

Pairwise matching, which is based on a measure (similarity or dissimilarity), is ubiquitous in multimedia applications. The performances of multimedia learning techniques depend sensitively on the selected measure [1–3]. Recently, measure learning has become an active research problem for multimedia learning tasks, for example, image classification [4, 5]. The previous measure learning studies mainly focused on distance (dissimilarity) learning. One of the earliest distance learning algorithms was presented by Xing et al. [6], where a parameterized Mahalanobis distance was learned. Many distance learning studies were followed [7–10], which would be overviewed later. There are two aspects of disadvantages for a distance metric. On the one hand, a distance learning task results in an optimization problem which is usually not easy to give a closed-form solution. Xing et al. [6], Lee et al. [11], Kumar and Kummamuru [12], Jin et al. [13], and Yin et al. [14] all described the distance metric learning through iterative process. The iterative methods are difficult to be extended to kernel versions. Moreover, the iterative procedure is inefficient and unstable. On the other hand, several recent studies suggest that the strict metric axioms (self-similarity, symmetry, and triangle inequality) are epistemologically invalid for perceptual distance of human beings [15, 16] and not so suitable for robust pattern recognition [17].

The other aspect of the relationship between two objects in learning tasks is similarity. Since the measure models vary in engineering practice, dissimilarity and similarity are not simply complementary. The similarity cannot be simply viewed as the negative or reciprocal dissimilarity. It is necessary to distinguish these two notions. The similarity measures include two categories: inner product based and kernel function based, which were both considered in this work.

Many publications support that the intrinsic structure of the feature space for image classification lies on low-dimensional manifolds [18–20]. Compared with Euclidean distance, correlation has some competitive abilities to capture the intrinsic structure embedded in the high-dimensional data. Correlation is a type of normalized inner product and a scale invariant index. It corresponds to the notion of “angle” in geometrical theory. In recent years, some studies have used correlation as a similarity measure for dimension reduction [21–23]. However, since correlation is in the fraction form, the existing correlation-based dimension reduction algorithms, such as correlation embedding analysis (CEA) [21], canonical correlation analysis (CCA) [22], and correlation discriminant analysis (CDA) [23], constructed low-dimensional embeddings through the iterative procedures.

In this work, we presented a similarity measure learning framework for supervised classification. Particularly, under this framework, a correlation similarity measure learning algorithm was constructed with a closed-form solution. It did not need iterative update process and is only involved in eigenvalue decomposition operations. Furthermore, it was extended into a kernelized version.

In order to learn an appropriate similarity measure, dissimilarity metric (distance) learning and dimensionality reduction can bring us much inspiration. Here, we provided a concise review on them.

### 1.1. Dissimilarity Metric Learning

Many dissimilarity metric learning algorithms have been presented in a variety of application areas. From the diverse points of view, these methods can be divided into different ways. Generally, there are two ways to categorize them: (1) unsupervised learning and supervised learning and (2) global method and local method. In this work, the latter categorization scheme is adopted.

For global methods, the well-known one is the earlier distance metric learning algorithm Xing et al. presented [6], which will be shown in detail later. This algorithm is further extended to the nonlinear case in [24] by the introduction of kernels, where a given kernel is idealized such that it becomes more similar to the ideal kernel also leads to a quadratic programming problem. Relevant component analysis (RCA) [25] learns a global linear transform from the equivalent constraints. Instead of iterative solution in [6], it only uses closed-form expressions of data and is based on subsets of points so-called chunklets. However, RCA has two important disadvantages. One is the lack of exploiting negative constraints which can also be informative, and the other is its incapability of capturing complex nonlinear relationships between data instances with the contextual information [8]. Discriminative component analysis (DCA) and kernel DCA [8] improve RCA by exploring negative constraints with contextual information. Kernel RCA [26] and kernel DCA use kernel trick to discover the nonlinear structures of the given data. Recently, Wang [9] proposed a method to learn Mahalanobis distance metric in semisupervised mode by maximizing the so-called constraint-margin maximization (CMM) criterion. CMM is based on graph embedding framework [27] often used in dimensionality reduction problems.

All the global methods are based on global constraints or side information. However, the real-world data may not satisfy the global linear assumption. So more approaches fall into local based category which approximates global nonlinear data structures based on local linear alignment. Discriminant adaptive nearest neighbor [10] estimates a local distance metric using the local linear discriminant analysis. Neighbourhood components analysis [28] learns a Mahalanobis distance metric by directly maximizing a stochastic variant of the leave-one-out KNN score on the training set. The maximum-margin nearest neighbor (LMNN) classifier [29] extends NCA through a maximum-margin framework. It reformulated the optimization problem as an instance of semidefinite programming, which was also solved by iterative process. Many other recent studies [29–31] also focus on neighbor information.

### 1.2. Dimensionality Reduction

Most algorithms above are based on so-called Mahalanobis distance function framework, which may be viewed as constructing a global linear transformation of the data and then applying the Euclidean distance over the transformed data. Mahalanobis distance is as follows:

(1) $$\begin{array}{c} d\left(x,y\right)={||x-y||}_{A}=\sqrt{{\left(x-y\right)}^{T}A\left(x-y\right)}. \end{array}$$

It requires *A*⪰0 to ensure that this can be used as a metric. So *A* can be represented as *A* = *WW* ^*T*^. Then to learn Mahalanobis distance is equivalent to finding a transform matrix *W* (*y* = *W* ^*T*^ *x*). Learning the transformation matrix *W* can yield the Mahalanobis metric *A* = *WW* ^*T*^ according to

(2) ||yi−yj||2=(xi−xj)TWWT(xi−xj)=(xi−xj)TA(xi−xj)=||xi−xj||A2.

For those dimensionality reduction methods without explicit transformation, they may also be viewed as searching appropriate embedding in a lower-dimensional space. So distance metric learning has an affinity with dimensionality reduction.

Methods on dimensionality reduction can be divided into two categories: (1) with explicit transformation and (2) with implicit transformation. The former includes almost all subspace learning algorithms. PCA, LDA, NMF, LPP [32], Laplacian Eigenmap [33], and their extended visions [34–36], all result in a transform matrix with optimizing some objective criterions. Most of classical manifold learning algorithms, such as LLE, ISOMAP, and LTSA, belong to the latter category. Since being without explicit transformation, manifold-based methods are more suitable for data visualization than classification. Inspired by NMF and LPP, graph embedding framework [27] becomes popular [37, 38]. It provides more flexibility through designing diverse graphs and weight matrices.

This paper aims at solving the following problem: given a set of sample data with class labels or pairwise constraints, the task is to learn an appropriate similarity measure for classification. In the beginning of this paper, several related distance learning and dimensionality reduction algorithms will be introduced. The previous work of correlation usage in classification will be also discussed in Section 2. The methods of Xing et al. and Xiang et al. are both used to learn the distance metric. However, Xing et al. mainly concentrated on clustering application. CEA [21], CCA [22], and CDA [23] all apply correlation for classification. Moreover, CEA [21] is also based on graph embedding framework [27] as our method does. These algorithms will be all described detailedly in Section 2.

Sections 3, 4, and 5 form the core of this paper.  Section 3 gives the precise definition of similarity measure learning problem and introduces a general formulation for it. This formulation can be specified to diverse measure learning algorithms depending on the determination of neighbor graph, affinity weights, and similarity measure. In Section 4, a strategy is given to form specific similarity measure learning algorithm. Firstly, a generalized correlation *ρ* is defined. After that, two kinds of constraints are introduced, which are based on two kinds of neighbor graphs and corresponding affinity weights. Most importantly, an approximate optimization and its closed-form solution are presented. Following that, it is extended to the nonlinear version in Section 5. Experiments have been conducted to prove the effectiveness of these new measure learning approaches for classification. They will be reported in Section 7. Additionally, discussions and conclusions will be given, respectively, in Sections 6 and 8.

The overall sequence of the core sections in this paper can be illustrated as follows.

*Similarity Measure Learning for Classification*

 SML—A framework
   The definition of SML problem
   General framework for SML
 CSML—An algorithm
   Generalized correlation *ρ*
   Optimization problem for CSML
   The closed-form solution of CSML
 KCSML—A nonlinear extension of CSML.

## 2. Related Work

This section provided a brief overview of closely related studies. From this analysis, our work would be placed in the context of other algorithms.

### 2.1. Xing and Xiang's Methods

Consider the form of a distance metric as follows:

(3) $$\begin{array}{c} d\left(x,y\right)={||x-y||}_{A}=\sqrt{{\left(x-y\right)}^{T}A\left(x-y\right)}, \end{array}$$

where *A*⪰0. Xing et al. introduced one of the earliest distance metric learning methods using both positive and negative constraints [6]. They posed distance metric learning as the following convex optimization problem:

(4) min⁡ J=∑(xi,xj)∈S||xi−xj||A2s.t. ∑(xi,xj)∈D||xi−xj||A≥1   A⪰0,

where *S* was the set of positive constraints and *D* was the set of negative constraints. The optimal metric was found by minimizing the distances between data points in affinity-link constraints and simultaneously maximizing the distances between data points in apart-link constraints. Xing et al. [6] used the gradient descent and the idea of iterative projection to solve the problem (4). Although the presented optimization problem was convex, it was a hard problem to solve. And the introduced solution in [6] was slow and somewhat unstable [8].

Xiang et al. [39] introduced the trace-ratio objective function (with the constraint *W* ^*T*^ *W* = *I*) as a more appropriate objective function:

(5) $$\begin{array}{c} W^{*}=\arg\;\underset{W^{T}W=I}{\max}\frac{\mathrm{tr}\left(W^{T}{\hat{S}}_{b}W\right)}{\mathrm{tr}\left(W^{T}{\hat{S}}_{w}W\right)}. \end{array}$$

However, this problem cannot be directly solved by eigenvalue decomposition approaches. To solve the problem (5), Xiang et al. [39] had constructed an iterative framework, in which a lower bound and an upper bound including the optimum were estimated for initialization. Their proposed method provides a heuristic search to solve the problem (5). In this work, we propose a generalized form of similarity measure learning rather than dissimilarity measure learning and provide a closed-form solution of objective function with correlation similarity.

### 2.2. CEA

Fu et al. [21] introduced correlation embedding analysis (CEA) for dimensionality reduction. Firstly, two undirected weighted graphs, the intrinsic graph *G* ^*I*^ = (*X*, *W* ^*I*^) and the penalty graph *G* ^*P*^ = (*X*, *W* ^*P*^), were constructed. *X* was a set of data vertexes and *W* ^*I*^, *W* ^*P*^ ∈ *R* ^*n*×*n*^ are weight matrices. The intrinsic graph characterizes data links that the algorithm favors and the penalty graph describes relationships that the algorithm tries to avoid. Then, a graph-preserving criterion is imposed for these two objectives as

(6) argmax⁡W{F(W)  =∑i≠j||WTxi||WTxi||−WTxj||WTxj||||2·(wijP−wijI)},

where *w*_*ij*_^*P*^ and *w*_*ij*_^*I*^ are the elements of weight matrices *W* ^*P*^ and *W* ^*I*^, respectively. It can be viewed as finding transformation matrix *W* in the linear transformation space of normalized samples. The formulation (6) can be rewritten as

(7) argmax⁡W{F(W) =2∑i≠j(1−xiTWWTxj(xiTWWTxi)(xjTWWTxj))    ·(wijP−wijI)}.

This objective function is nonlinear and not convex. Fu et al. [21] used the gradient descent rule for optimization by differentiating *F*(*W*) with respect to matrix* W*. As pointed in [21], the gradient descent may not be deep enough to converge to a good solution when the dimension of the data space is too large. So the iterative process is sensitive on the initial point although the method to find a good initialization was proposed. In this paper, we transform the problem (7) into another optimization problem which can be solved with closed-form solution.

### 2.3. Correlation in Classification

Next, we will focus on the usage of correlation in classification. Hardoon et al. [22] introduced canonical correlation analysis (CCA). It can be viewed as the problem of finding basis vectors for two sets of variables such that the correlations between the projections of the variables are mutually maximized. If one set of variables is taken as class labels, CCA can be used to realize a supervised linear feature extraction and subsequent classification. It has been extended to a nonlinear version kernel CCA by kernel trick. However, there are some problems when it is used in classification application as pointed in [23], which limits its utilization in practice.

Ma et al. [23] introduced correlation discriminant analysis (CDA) which sought a global linear transformation to maximize the correlation of samples from different classes in the transformed space. Its optimization problem was

(8)  max⁡A (Sw−Sb)or  max⁡A (Sw−St)  s.t.  A⪰0,

where

(9) Sw=1Nw∑ti=tjyiTyj||yi||||yj||‍=1Nw∑ti=tjxiTwTwxjxiTwTwxixjTwTwxj,‍Sb=1Nw∑ti≠tjyiTyj||yi||||yj||‍=1Nw∑ti≠tjxiTwTwxjxiTwTwxixjTwTwxj,‍St=1Nw+Nb∑ti,tjyiTyj||yi||||yj||=1Nw∑ti,tjxiTwTwxjxiTwTwxixjTwTwxj‍A=WTW.

In [23], this problem was also solved by gradient-based optimization method. However, the extension of CDA to kernel CDA was not very easy to be implemented, as pointed in [23].

## 3. General Framework

Similarity measure learning (SML) is a general framework for similarity measure learning problem. In the context of general supervised classification, the SML problem may be formulated as follows: given a labeled sample set {(*X*, *Y*)}, with *n* instances, {*x*_*i*_}_*i*=1_^*n*^ ∈ *R* ^*D*^, and *D* is the feature dimension. The corresponding class label is {*y*_*i*_}_*i*=1_^*n*^ ∈ {1,…, *c*}, where *c* is the number of classes. Suppose that the similarity measure between arbitrary two objects *x*_*i*_ and *x*_*j*_ is *ρ*(*x*_*i*_, *x*_*j*_, *W*), where *W* is a set of parameters to be learned. The goal of SML is to learn the parameter set *W* from the sample set {(*X*, *Y*)}.

We now introduce SML problem from the novel point of view of graph embedding. Let *G* = {(*X*, Δ)} be an undirected weighted graph with vertex set *X* and relation matrix Δ ∈ *R* ^*n*×*n*^. We define an intrinsic graph *G* ^*I*^ = {(*X*, Δ^*I*^)}, where Δ^*I*^ = [*δ*_*ij*_^*I*^]_*n*×*n*_, and a penalty graph *G* ^*P*^ = {(*X*, Δ^*P*^)}, where Δ^*P*^ = [*δ*_*ij*_^*P*^]_*n*×*n*_. Vertices *X* of graph *G* ^*I*^ are the same as those of graph *G* ^*P*^, but the matrix Δ^*I*^ corresponds to the relations that are to be strengthened and the matrix Δ^*P*^ corresponds to the relations that are to be suppressed in the learning process.

Based on the above evidences, we get the formal definition of the similarity measure learning.

**Definition 1 .**

The similarity measure learning (SML) problem is to learn an optimal similarity measure [*ρ*_*ij*_]_*n*×*n*_ from a collection of data points *X* on a vector space *R* ^*D*^ together with a set of intrinsic pairwise constraints *G* ^*I*^ and a set of penalty pairwise constraints *G* ^*P*^, which can be formally formulated into the following optimization framework:

(10) $$\begin{array}{c} \min\left(\text{or}\;\;\max\right)f\left(W,G^{I},G^{P},X\right), \end{array}$$

where *W* is a set of parameters to be learned and *f* is some objective function defined over the given data.

Inspired by graph embedding learning in dimensionality reduction, SML can be formulated as the following two objectives based on graph-preserving criterion:

(11) max⁡W∑i≠jρ2(xi,xj,W)δijImin⁡W∑i≠jρ2(xi,xj,W)δijP.

To combine these two objectives into a unique optimization problem, there exist several different ways [27]. In this work, we consider the difference-form formulation; namely,

(12) $$\begin{array}{c} W^{*}=\arg\;\max\left\{L\left(W\right)=\underset{i\neq j}{\sum }\rho^{2}\left(x_{i},x_{j},W\right)\left(\delta_{ij}^{I}-\delta_{ij}^{P}\right)\right\}. \end{array}$$

It can be seen from Definition 1 that the method proposed in the next section will be also suitable for classification problem with pairwise constraints instead of labels.

## 4. Correlation Similarity Measure Learning

In this section, we introduce a generalized correlation measure *ρ*. Based on the generalized correlation, an algorithm of SML, called correlation similarity measure learning (CSML), is proposed. It aims at learning a correlation similarity measure for classification. The details are summarized in Algorithm 1.

### 4.1. Objective Function

“Correlation” is one of widely used measures to reflect the similarity between two random variables. Correlation is also termed as normalized correlation, correlation coefficient, Pearson's correlation, or cosine similarity, and hereafter correlation for simplicity. Two samples (e.g., images) are represented as two vectors *x*_*i*_ and *x*_*j*_ in a feature space, and then the standard form of correlation is

(13) $$\begin{array}{c} \underset{}{\text{corr}}\left(x_{i},x_{j}\right)=\frac{x_{i}^{T}x_{j}}{\sqrt{x_{i}^{T}x_{i}}\sqrt{x_{j}^{T}x_{j}}}. \end{array}$$

In learning tasks, to make the similarity measure flexible to sample data, we define a generalized correlation.

**Definition 2 .**

The generalized correlation of random vectors *x*_*i*_ and *x*_*j*_ is defined as

(14) $$\begin{array}{c} \rho \left(x_{i},x_{j}\right)=\frac{x_{i}^{T}Mx_{j}}{\sqrt{x_{i}^{T}Mx_{i}}\sqrt{x_{j}^{T}Mx_{j}}}, \end{array}$$

where *M* ∈ *R* ^*D*×*D*^ is a parameter matrix and symmetric positive semidefinite; for example, *M*⪰0.

So, in the paper, let *M* = *WW* ^*T*^, where *W* = [*w*_1_, *w*_2_,…, *w*_*d*_] ∈ *R* ^*D*×*d*^ and *d* is an alternative parameter. Generally, matrix *M* parameterizes a family of the correlations on the vector space *R* ^*D*^. Specifically, when *M* is an identity matrix *I*_*D*×*D*_, the generalized correlation in (14) becomes the standard correlation.

This type of correlation measure assigns different importance on series of features rather than equally processing as standard correlation coefficient does. It enhances the flexibility of the similarity measure. The parameter matrix *M* could be adaptive for sample data.

Equation (14) can be modified to its equivalent form as

(15) $$\begin{array}{c} \rho \left(x_{i},x_{j},W\right)=\frac{\mathrm{tr}\left(W^{T}x_{i}^{T}x_{j}W\right)}{\sqrt{\mathrm{tr}\left(W^{T}x_{i}^{T}x_{i}W\right)}\sqrt{\mathrm{tr}\left(W^{T}x_{j}^{T}x_{j}W\right)}}, \end{array}$$

where tr⁡(·) denotes the trace of a matrix.

Substitute (15) into the optimization problem (12) and then we obtain the objective function as follows:

(16) L(W)=∑i≠jtr⁡(WTxixjTW)tr⁡(WTxixiTW)tr⁡(WTxjxjW)‍   ·(δijI−δijP).

### 4.2. Intrinsic Graph and Penalty Graph

Intrinsic graph *G* ^*I*^ and penalty graph *G* ^*P*^ are both structural representations based on pairwise object comparisons. For such a representation, class overlap does not exist if the objects can be unambiguously labeled; there are no real-world objects in the application which belong to more than one class. Moreover, this structural representation can utilize the prior knowledge or supervised information in an alternative way, which will be discussed later.

#### 4.2.1. Global Constraints

For *G* ^*I*^, the node *x*_*i*_ and the node *x*_*j*_ are connected by an edge if *x*_*i*_ and *x*_*j*_ belong to the same class, otherwise not connected. For *G* ^*P*^, the edge between *x*_*i*_ and *x*_*j*_ is constructed if *x*_*i*_ and *x*_*j*_ belong to different classes, otherwise not constructed. In our experiments, the global scheme is adopted.

#### 4.2.2. Local Constraints

For *G* ^*I*^, only consider each pair of *x*_*i*_ and *x*_*j*_ from the same class. The node *x*_*i*_ and the node *x*_*j*_ are connected if *x*_*j*_ is among the most nearest *k* ^*I*^ nodes from *x*_*i*_ or *x*_*j*_ is in the circle neighbor region *ɛ*_*γ*_^*I*^ of *x*_*i*_. This is based on neighbor Euclidean distance. For *G* ^*P*^, only consider each pair of *x*_*i*_ and *x*_*j*_ from the different classes. The edge between *x*_*i*_ and *x*_*j*_ is constructed if *x*_*j*_ is among the most nearest *k* ^*P*^ nodes from *x*_*i*_ or *x*_*j*_ is in the circle neighbor region *ɛ*_*γ*_^*P*^ of *x*_*i*_. Here, *k* ^*I*^, *ɛ*_*γ*_^*I*^, *k* ^*P*^, and *ɛ*_*γ*_^*P*^ are all alternative parameters. It is obvious that the local scheme is appropriate for unsupervised learning and semisupervised learning.

### 4.3. Affinity Weights

After determining the edge distribution of constraint graph, it needs to assign affinity weights to the edges. Suppose *d*(*x*_*i*_, *x*_*j*_) = (*x*_*i*_ − *x*_*j*_)^*T*^(*x*_*i*_ − *x*_*j*_) is the distance between *x*_*i*_ and *x*_*j*_. Following existing work, here we have two variations for weighting the edges: (1) Gaussian kernel or other kernels: *δ*_*ij*_ = *e* ^−*d*^2^(*x*_*i*_, *x*_*j*_)/*t*^ and (2) simple-minded: *δ*_*ij*_ = 1, if *i* and *j* are connected; otherwise, *δ*_*ij*_ = 0. These two ways have respective advantages and shortages. Δ^*I*^ and Δ^*P*^ can choose different weighting schemes and parameters. It provides the flexibility in practical application. The latter scheme is adopted in our method.

### 4.4. Closed-Form Solution for CSML

Motivated by [40], in this section, we will give a closed-form solution for SML to avoid the iterative optimization over high-dimensional space. For the optimization problem (12), additionally, we introduce an orthogonal constraint; that is, *w*_*i*_^*T*^ *w*_*j*_ = 0, for all *i* ≠ *j*. The problem (12) may be transformed into the following maximum optimization:

(17) max⁡ J=∑i≠jtr⁡(WTxixjTW)δij(I−P)‍s.t. tr⁡(WTxixiTW)=1, ∀iwiTwj=0, ∀i≠j,

where *δ*_*ij*_^(*I*−*P*)^ is short for *δ*_*ij*_^*I*^ − *δ*_*ij*_^*P*^. For simplicity, introduce the matrix notation

(18) $$\begin{array}{c} S_{\delta }=\underset{i\neq j}{\sum }\delta_{ij}^{\left(I-P\right)}x_{i}x_{j}^{T}. \end{array}$$

In fact, *S*_*δ*_ is a weighted sum of covariance matrices of sample data. Next, *w*_*i*_ will be computed, respectively. To obtain the best discriminant vector *w*_1_, we introduce the following Lagrange function with multipliers *λ*_*i*_:

(19) JL1=w1TSδw1−λ1(w1Tx1x1Tw1−1) −⋯−λn(w1TxnxnTw1−1).

Considering *λ*_1_ = *λ*_2_ = ⋯ = *λ*_*n*_ = *λ*, compute the partial derivative of *J*_*L*1_ with respect to *w*_1_ and set it to zero; then

(20) $$\begin{array}{c} S_{\delta }w_{1}=\lambda S_{e}w_{1}, \end{array}$$

where

(21) $$\begin{array}{c} S_{e}=\overset{m}{\underset{i=1}{\sum }}x_{i}x_{i}^{T}. \end{array}$$

Here, *w*_1_ is the eigenvector of *S*_*e*_^−1^ *S*_*δ*_ associated with the largest eigenvalue.

To obtain other *w*_*i*_, we introduce the following Lagrange function with multipliers *λ* and *μ*_*i*_:

(22) JL=∑l=1mwlTSδwl−λ‍(∑l=1mwlTSewl−n‍)−μ1wmTw1  −μ2wmTw2−⋯−μm−1wmTwm−1.

*w*_*m*_ can be obtained by maximizing the above Lagrange function. As the above process, compute the partial derivative of *J*_*L*_ with respect to *w*_*m*_ and set it to zero:

(23) ∂JL∂wm=2Sδwm−2λSewm−μ1w1−μ2w2 −⋯−μm−1wm−1=0.

Multiply the two sides of (23) by *w*_*m*_^*T*^; then

(24) $$\begin{array}{c} \lambda =\frac{w_{m}^{T}S_{\delta }w_{m}}{w_{m}^{T}S_{e}w_{m}}. \end{array}$$

Thus *λ* represents the expression to be maximized. Considering (23), multiply its two sides successively by *w*_1_^*T*^ *S*_*e*_^−1^,…, *w*_*m*_^*T*^ *S*_*e*_^−1^, and then obtain *m* − 1 equations:

(25) μ1w1TSe−1w1+⋯+μm−1w1TSe−1wm−1 =2w1TSe−1Sδwm,μ1w2TSe−1w1+⋯+μm−1w2TSe−1wm−1 =2w2TSe−1Sδwm,  ⋮μ1wm−1TSe−1w1+⋯+μm−1wm−1TSe−1wm−1 =2wm−1TSe−1Sδwm.

If we use matrix notations,

(26) μm−1=[μ1,μ2,…,μm−1]T,Wm−1=[w1,w2,…,wm−1],Dm−1=[Dijm−1]=[Wm−1]TSe−1Wm−1,Dijm−1=wiTSe−1wj.

The previous set of (*m* − 1) equations can be represented in a single matrix relationship:

(27) $$\begin{array}{c} D^{m-1}\mu^{m-1}=2{\left[W^{-1}\right]}^{T}S_{e}^{-1}S_{\delta }w_{m} \end{array}$$

or in another form

(28) $$\begin{array}{c} \mu^{m-1}=2{\left[D^{m-1}\right]}^{-1}{\left[W^{-1}\right]}^{T}S_{e}^{-1}S_{\delta }w_{m}. \end{array}$$

Let us multiply the two sides of (23) by *S*_*e*_^−1^:

(29) 2Se−1Sδwm−2λwm−μ1Se−1w1−μ2Se−1w2 −⋯−μm−1Se−1wm−1=0.

This can be expressed using matrix notation as

(30) $$\begin{array}{c} 2S_{e}^{-1}S_{\delta }w_{m}-2\lambda w_{m}-S_{e}^{-1}{\left[W^{m-1}\right]}^{T}\mu^{m-1}=0. \end{array}$$

Including (28), we have

(31) $$\begin{array}{c} Lw_{m}=\lambda w_{m}, \end{array}$$

where *L* = (*I* − *S*_*e*_^−1^[*W* ^*m*−1^]^*T*^[*D* ^*m*−1^]^−1^ *W* ^*m*−1^)*S*_*e*_^−1^ *S*_*δ*_. Considering *λ* as the criterion to be maximized, *w*_*m*_ is the eigenvector of *L* and is associated with the largest eigenvalue of *L*.

### 4.5. Singularity of *S*_*e*_

To model the similarity measure, it only needs to obtain the *d* largest eigenvalues of *L* to constitute *W* = [*w*_1_,…, *w*_*d*_]. However, involving with the inverse of *S*_*e*_, it cannot be applied when *S*_*e*_ is singular due to the small sample size problem. The small sample size problem occurs frequently in practice. In many applications, the dimensionality of the sample features is extraordinarily high while the number of samples is much small in comparison. When the number of samples is smaller than that of features, the small sample size problem occurs, for example, face recognition, text document classification, image retrieval, and cancer classification with gene expression profiling. The dimensionality of input space is high while the sample is often lacking. To handle this problem, the direct method is to replace *S*_*e*_^−1^ with the pseudoinverse matrix *S*_*e*_^†^. However, it does not guarantee that graph-preserving criterion is still optimized by the largest eigenvectors involved with *S*_*e*_^†^. Here, the problem is similar to that in LDA. For the singularity in LDA, there are several frequently used methods, which can be modified for SML. The common way is to add a singular value perturbation to *S*_*w*_ to make it nonsingular [41]. Null subspace method and direct LDA [42] are both well known. Another one is kernel Fisher's discriminant (KFD), which is a nonlinear extension to LDA. Maximum margin criterion (MMC) [43] modified the criterion in the fraction form into a difference one, which avoids the small sample size. In this work, we first employ PCA to reduce the dimensionality of the feature space to *n* − 1, where *n* is the number of samples and then apply SML on the dimensionality-reduced subspace.

## 5. Kernel CSML

CSML is used to find a global linear transformation matrix although the graph with local constraints may capture local nonlinear properties. In many cases, kernel trick is an efficient technique to extend a linear method to its nonlinear version.

To perform our linear method in reproducing kernel Hilbert space (RKHS), we consider the problem in a feature space *F* induced by a nonlinear mapping *ϕ* : *R* ^*n*^ → *F*. We can define a Mercer's kernel function: *k*(*x*, *y*) = 〈*ϕ*(*x*), *ϕ*(*y*)〉 = *ϕ* ^*T*^(*x*)*ϕ*(*y*), where *k*(·, ·) is a positive semidefinite kernel.

In the feature space *F*, the generalized correlation similarity measure has the form

(32) ρ(ϕ(xi),ϕ(xj),W) =ϕT(xi)WWTϕ(xj)ϕT(xi)WWTϕ(xi)ϕT(xi)WWTϕ(xj).

Of course, it has the equivalent form

(33) ρ(ϕ(xi),ϕ(xj),W) =tr⁡(WTϕ(xi)ϕT(xj)W)tr⁡(WTϕ(xi)ϕT(xi)W)tr⁡(WTϕ(xj)ϕT(xj)W).

Since, in the feature space *F*, *W* lies in the linear combination of *ϕ*(*x*_1_), *ϕ*(*x*_2_),…, *ϕ*(*x*_*n*_), it can be defined as

(34) $$\begin{array}{c} W=\Phi \left(X\right)\alpha , \end{array}$$

where Φ(*X*) = [*ϕ*(*x*_1_), *ϕ*(*x*_2_),…, *ϕ*(*x*_*n*_)] represents the training data in feature space *F* and *α* = [*α*_*ij*_]_*n*×*d*_. Specially, *α*_*j*_ = [*α*_1*j*_, *α*_2*j*_,…, *α*_*nj*_]^*T*^ and *w*_*j*_ = ∑_*i*=1_^*n*^ *α*_*ij*_ *ϕ*(*x*_*i*_) = Φ(*X*)*α*_*j*_. Substitute (33) and (34) into (17) and obtain

(35) max⁡ J=∑i≠jtr⁡(αTΦ(X)Tϕ(xi)ϕ(xj)TΦ(X)α)δij(I−P)s.t. tr⁡(αTΦ(X)Tϕ(xi)ϕ(xi)TΦ(X)α)=1, ∀i   αiTΦ(X)TΦ(X)αj=0, ∀i≠j.

Define kernel matrix as

(36) $$\begin{array}{c} K={\left[k\left(x_{i},x_{j}\right)\right]}_{nn}={\left[k_{ij}\right]}_{nn}. \end{array}$$

Let

(37) Sδk=∑i≠j‍δijΦ(X)Tϕ(xi)ϕ(xj)TΦ(X)=KΔK,Sek=∑i=1nΦ(X)Tϕ(xi)ϕ(xi)TΦ(X)=KK,Sk=Φ(X)TΦ(X)=K.

The following Lagrange function with multipliers *λ* and *μ*_*i*_ is introduced:

(38) JL=∑l=1dαlTSδkαl−λ(∑l=1dαlTSekαl−n)‍  −μ1αmTα1−μ2αmTα2−⋯−μm−1αmTαm−1.

Comparing with the analysis of CSML, some notations are introduced:

(39) μm−1=[μ1,μ2,…,μm−1]T,αm−1=[α1,α2,…,αm−1],Bm−1=[Bijm−1]=[αm−1]T(Sek)−1Skαm−1,Bijm−1=αiT(Sek)−1Skαj.

Note that the above notations are a little different from (26). Similarly, the final solution is obtained: *α*_1_ is the largest eigenvector of *S*_*e*_^*k*^ ^−1^ · *S*_*δ*_^*k*^ and *α*_*m*_ is the largest eigenvector of the matrix

(40) $$\begin{array}{c} \left(I-{\left(S_{e}^{k}\right)}^{-1}S^{k}\left[\alpha^{m-1}\right]{\left[B^{m-1}\right]}^{-1}{\left[\alpha^{m-1}\right]}^{T}\right){\left(S_{e}^{k}\right)}^{-1}S_{\delta }^{k}. \end{array}$$

Here, we note that the problem of the eigenvalue decomposition of (40) is ill-posed because the rank of the square matrix *S*_*e*_^*k*^ is less than or equal to *n* − 1 and then *S*_*e*_^*k*^ is singular. To handle the singularity of *S*_*e*_^*k*^, we simply add a small positive perturbation to *S*_*e*_^*k*^, that is, replay *S*_*e*_^*k*^ by ${\bar{S}}_{e}^{k}$, where

(41) $$\begin{array}{c} {\bar{S}}_{e}^{k}=S_{e}^{k}+\mu I. \end{array}$$

We set *μ* = 10^−3^ in this work.

## 6. Discussion

### 6.1. The Trace-Ratio, Ratio-Trace, and Trace-Difference

It is known that the trace-ratio optimization problem is nonconvex and has no closed-form solution. CSML is the typical one of this type of problem. To solve such a problem, there have been some attempts. The most popular is to transform such problems into the ratio-trace problem. For (16), the corresponding ratio-trace form is

(42) $$\begin{array}{c} \tilde{L}\left(W\right)=\underset{i\neq j}{\sum }\mathrm{tr}\left(\frac{W^{T}x_{i}x_{j}^{T}W}{\sqrt{W^{T}x_{i}x_{i}^{T}W}\sqrt{W^{T}x_{j}x_{j}W}}\right)\left(\delta_{ij}^{I}-\delta_{ij}^{P}\right), \end{array}$$

which can be approximately solved with the general eigenvalue decomposition (GEVD) method:

(43) $$\begin{array}{c} S_{\delta }w_{k}=\tau_{k}S_{e}w_{k}, \end{array}$$

where *τ*_*k*_ is the *k*th largest eigenvalue of the GEVD associating with the eigenvector *w*_*k*_ and *w*_*k*_ constitutes the *k*th column vector of the matrix *W*. Finally, *M* = *WW* ^*T*^ and the measure is learned. It can be seen that it is a suboptimal solution of the optimal problem (17) proposed in this paper. As pointed in [44], despite the existence of a closed-form solution for ratio-trace optimization problem, its approximation may sacrifice the potential classification capability of the derived low-dimensional feature spaces and is unstable for supervised classification. Guo et al. [45] converted such trace-ratio problem to a trace-difference one. However, it is solved by the iterative algorithm. For the detailed analysis on these attempts, we will refer the readers to the prior work [44, 45].

In this work, an alternative approximate optimization problem and its solution are presented. The denominator of the original trace-ratio objective function is fixed and then the numerator is maximized alone. In fact, the problem (16) can be approximated to the trace-difference one as follows:

(44) $$\begin{array}{c} \hat{L}\left(W\right)=\mathrm{tr}\left(W^{T}\left(S_{\delta }-\lambda S_{e}\right)W\right), \end{array}$$

which is the same as the objective function in [45]. However, the following operations of CSML are very different from those in [45]. In CSML it just involves the eigenvalue decomposition, which is more simple and comprehensible.

### 6.2. Computational Complexity

The computational cost of CSML mainly comes from two parts. The first part is graph construction, that is, connecting each sample with its nearest neighbors, and its computational cost is *O*(*n* ^2^ *D*). The next part is the matrix eigenvalue decomposition, and its computational cost is *O*(*dD* ^3^). So the overall cost is *O*(*dD* ^3^ + *n* ^2^ *D*). For comparison, Table 1 illustrates the computational costs of several distance metric learning and dimensionality reductions related to CSML, where *T* is the number of iterations. We can see that Xing's method is most expensive on computational cost. Our approaches CSML and KCSML are both more efficient than other several related algorithms.

## 7. Experiments

To evaluate proposed algorithms CSML and KCSML, in this section, we perform several image classification experiments on diverse databases and compare them with another popular related work. These comparable methods include principal component analysis (PCA), random subspace two-dimensional PCA (RS-2DPCA), linear discriminant analysis (LDA), local preserving projection (LPP), marginal fisher analysis (MFA) [27], correlation embedding analysis (CEA), correlation discriminant analysis (CDA), improved similarity measure-based graph embedding (ISM-GE) [46], and maximal similarity embedding (MSE) [47]. PCA is taken as a baseline method. RS-2DPCA stands for the state of the art of unsupervised dimensionality reduction technique. LDA is a basic supervised discriminant technique. LPP and MFA stand for the state of the art of dimensionality reduction technique. CEA and CDA use standard correlation as their measure and closest to our method. Particularly, CEA is also designed in the graph-preserving framework. ISM-GE and MSE are both the most recent achievements of embedding learning based on the correlation metric, which are close to our method. ISM-GE defines a new improved similarity measure by fusing the Euclidean metric and the correlation metric and then performs graph embedding learning with the new measure. MSE searches for global linear dimensional reduction directions which preserve the local pairwise correlation similarity.

**Figure 1:**
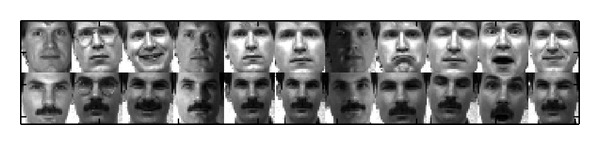
Samples from the Yale database.

**Figure 2:**
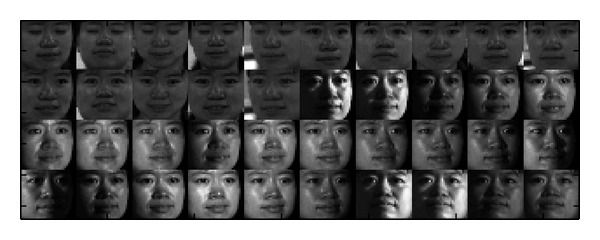
Samples from the CMU PIE database.

**Figure 3:**
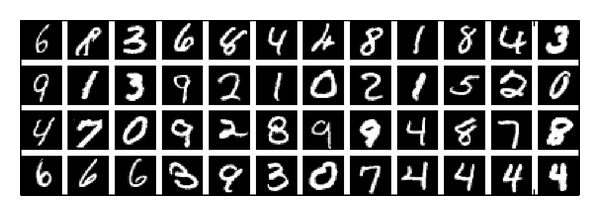
Samples from the MNIST database.

Face recognition is the classical application of image classification, which depends critically on a measure. The face databases Yale [48] and CMU PIE [49] are adopted. The MNIST is a popular handwritten digits database. We choose a subset from it as our experimental database. The image samples from the three databases are shown in Figures 1, 2, and 3, respectively. All the methods in experiments use centering and normalization as their preprocessing. The final classification is based on the simple nearest neighbor (NN) classifier. In all experiments, Gaussian kernel is adopted and the kernel width is set to the standard variance $\sigma =\text{sqrt}(\sum_{j=1}^{n}{||x_{j}-\bar{x}||}^{2}/n)$. All of the results reported for those algorithms in comparison are from the best tuning of their parameters. *d* in the table denotes the projection dimension when the best performance is got in PCA, RS-2DPCA, LDA, LPP, MFA, CDA, CEA, ISM-GE, and MSE. For CSML and KCSML, *d* denotes the number of largest eigenvectors to constitute the parameter matrix *W* for CSML and the matrix *α* for KCSML, respectively.

### 7.1. Classification on the Yale Database

The Yale Face Database contains 165 grayscale images of 15 individuals. There are 11 images per subject, varying on facial expression and configuration: center-light, w/glasses, happy, left-light, w/no glasses, normal, right-light, sad, sleepy, surprised, and wink. The images are cropped and resized into 32 × 32 pixels. The feature of each image is represented by a 1,024-dimensional column vector. A random subset with *p* images per individual is taken with labels to form the training set, where *p* = 2,3, 4,5, 6,7. The rest of the database is considered to be the testing set. For each given *p*, 50 randomly splits are constituted. The results reported in Table 2 are the average values for 50 splits.

From comparisons in Table 2, we can observe that all the supervised methods outperform the unsupervised method PCA. It is easy to understand it since more class label information is introduced. We also see that CSML and KCSML both outperform the other competitive methods under all configurations, particularly, no matter being with sufficient or insufficient quantity of sample data. It confirms that the proposed generalized correlation similarity measure can effectively capture the intrinsic affinity structure of the data. The more experimental results in [21] show that PCA, LDA, and LPP perform better based on the correlation NN classifier. In our experiments, the correlation based methods (CDA, CEA, CSML, and KCSML) outperform the other methods based on Euclidean distance. In most cases, the kernel extension of CSML is better than its original version. From these results, it is obvious that the similarity measure is more effective in recognition tasks than Euclidean distance.

### 7.2. Classification on the CMU PIE Database

The CMU PIE database contains 41,368 images of 68 people, each person under different poses, illumination conditions, and expressions. We select a subset, which contains images under five near frontal poses, different illuminations, and expressions. There are 170 images for each individual and 11,554 images in all. The images are cropped and resized to be 32 × 32 pixels. As processed in the former experiment, each image is unfolded as a column vector. A random subset with *p* = 5,10,20,30 images per individual is taken to form the training samples. The results in Table 3 are also average results of 50 splits for each *p*.

From Table 3, CSML and KCSML greatly outperform the other competitive methods. PCA still performs worst. The selected subset used in the experiment contains more than 10 thousand images. From the experimental results in Table 3, we can conclude that the proposed similarity measure is effective and reliable on large scale databases. It further demonstrates the ability of the generalized correlation measure to capture the intrinsic structure of high-dimensional data.

### 7.3. Classification on the MNIST Database

The MNIST database consists of 60,000 handwritten digit images from the larger database NIST. We select randomly 500 images for each digit and then 5000 images in total from MNIST, to constitute a smaller subset as our experimental database. The images have been normalized into 28 × 28 pixels. The feature of each digit is represented by a 784-dimensional vector. As processed in the former experiments, the subset with *p* = 50,100,150,200,250,300 images per digit was taken to form the training sample set. And the all left images are taken as testing samples for each training subset. Also, for each *p*, 50 random splits are constituted. The results in Table 4 are also average results of 50 splits for each *p*.

With the experimental results in Table 4, the similar conclusion can be obtained.

### 7.4. Effects of Parameter Selection

In our proposed algorithm, the *k*-nearest neighbor search is twice applied. The first one is used for affinity graph construction in terms of the local constraints. *k* ^*I*^ for intrinsic graph and *k* ^*P*^ for penalty graph can be different and chosen with empirical values. Here, we assume *k* ^*I*^ = *k* ^*P*^ = *k*_1_ to simplify the analysis. In the above experiments, we adopt the global scheme to construct pairwise affinity graphs (intrinsic graph and penalty graph) avoiding tedious tuning work. The other one, denoted by *k*_2_-NN, is used as the final classifier. For fairness, in the above experiments, all the compared methods uniformly use the same simple 1-nearest neighbor as the final classifier. In this subsection, to show more details of our proposed algorithm, we analyze the effects of these two types of parameters on the recognition performance.

**Figure 4:**
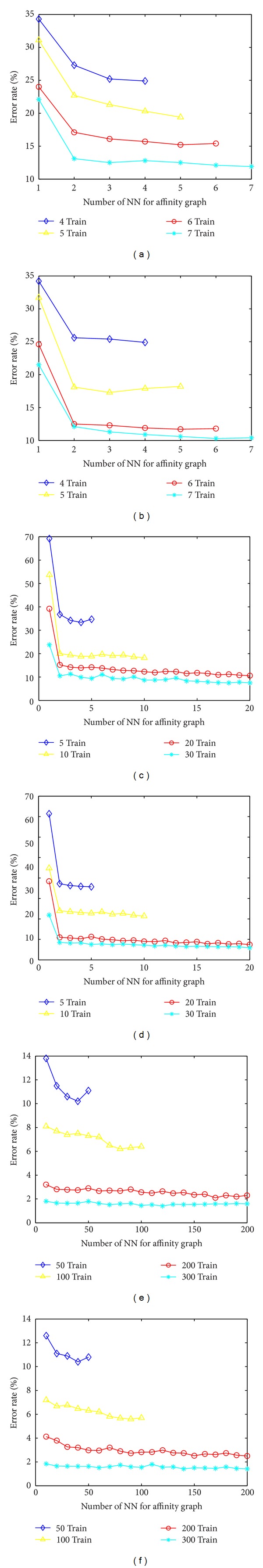
The behavior of the proposed methods under various *k*_1_. (a) CSML on Yale database, (b) KCSML on Yale database, (c) CSML on CMU PIE database, (d) KCSML on CMU PIE database, (e) CSML on MNIST database, and (f) KCSML on MNIST database.

Figure 4 shows the error rate variations of CSML and KCSML with different *k*_1_ on the three databases. The corresponding *d*'s are set to be the selected best ones in the above corresponding experiments. We find that the recognition performances of our proposed methods have a similar trend, where the error rate becomes approximately stable when the *k*_1_ are relatively large. This result confirms our intuition that the larger *k*_1_ covers the more constraints which are beneficial to the description of embedded relations. So, in practice, we suggest to choose a relatively larger *k*_1_, which is also the reason why we choose the global scheme for affinity graphs construction in the above comparison experiments.

**Figure 5:**
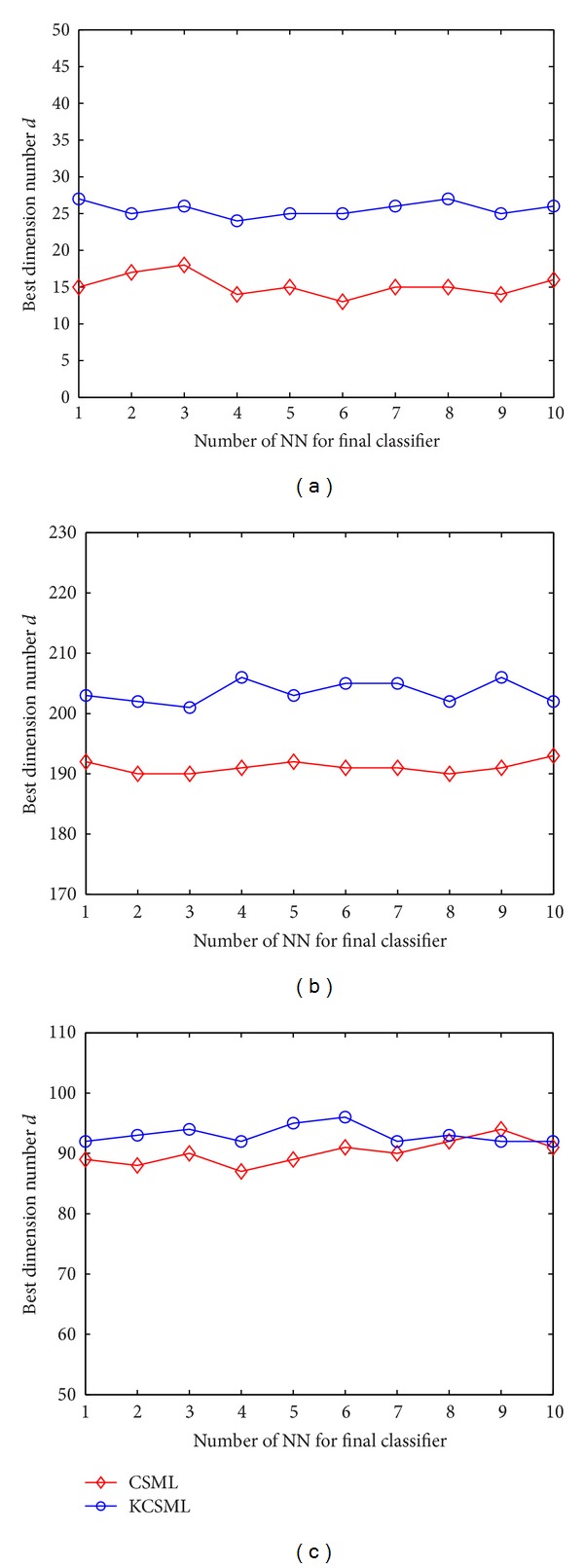
The best dimension number *d* for various *k*_2_: (a) on Yale database, (b) on CMU PIE database, and (c) on MNIST database.

Figure 5 shows the best dimension number *d*'s under different *k*_2_ on the Yale database with “7 Train,” the CMU PIE with “30 Train,” and the MNIST with “300 Train.” We find that the *d*'s have a very small variation with the changing of the *k*_2_; that is, we can choose a similar *d* for different *k*_2_. This result suggests that, for a given dataset, its intrinsic dimension number is determined no matter which classifier is selected. However, this has no benefit to parameter selection in practice. Generally, the parameter *d* is set through time-consuming cross-validation tests with empirical experiences. In the above experiments, all the parameters *d*'s are chosen by the threefold cross-validation tests in the empirical value ranges.

### 7.5. Executive Time

**Figure 6:**
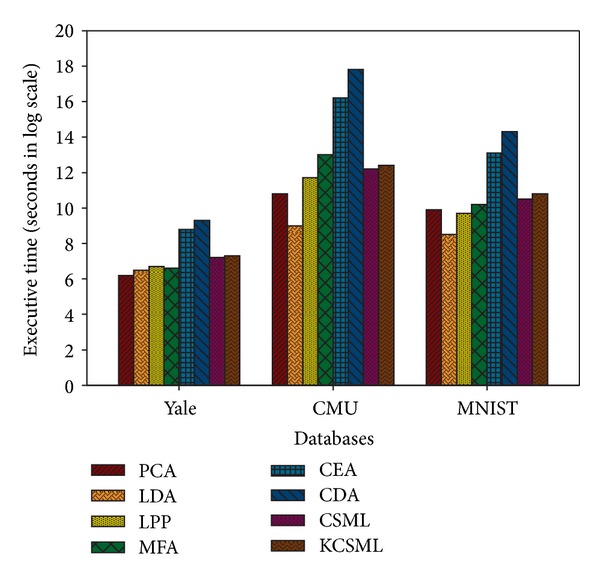
Comparison on CPU time in log scale.

In our experiments, we also consider the comparison of computational efficiency of these algorithms. The CPU times of these methods are executed for fifty runs on Yale with *p* = 7, CMU with *p* = 30, and MNIST with *p* = 300. The result in log scale is summarized in Figure 6. It shows that the executive times of CSML and KCSML are closest to each other and both are far less than those of CEA and CDA, which have comparable recognition rates with our presented methods. It agrees with the theoretical analysis result in Table 1. We could conclude that our presented methods are more efficient than CEA and CDA.

## 8. Conclusion

In this paper, we have presented a general framework for similarity measure learning (SML). The proposed generalized correlation *ρ* improves the flexibility of standard correlation. Based on the generalized correlation, a specific algorithm of SML, called CSML, and its kernel extension KCSML are proposed. Their objective functions are in trace-ratio form, which have no closed-form optimal solution. We transform the two objective functions to their approximate optimization problems and give their closed-form solutions. The experiments on face recognition database and handwritten digits database indicate that the proposed similarity measure is effective to capture the intrinsic affinity structure of high-dimensional data. Because of the flexibility of this general framework, the CSML can also be modified to the semisupervised version under local or global constraint information, which is not contained in this paper. Other experiments critically depending on the adopted measure, such as clustering and image retrieval, will be performed as the future work.

## Figures and Tables

**Algorithm 1:**
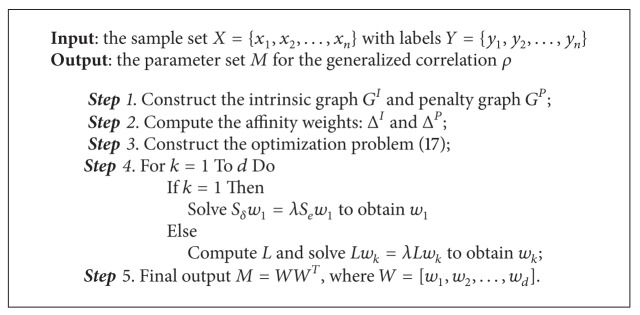
The details of algorithm CSML.

**Table 1:** Computational costs of related algorithms.

Algorithm	Computational cost
The method of Xing et al. (Xing's)	*O*(*D* ^6^)
The method of Xiang et al. (Xiang's)	*O*(*TD* ^4^)
Correlation Discriminant Analysis (CDA)	*O*(*T*(*D* ^4^ + *D* ^2^ *n* ^3^))
Correlation Embedding Analysis (CEA)	*O*(*T*(*D* ^4^ + *D* ^2^ *n* ^3^))
Correlation Similarity Measure Learning (CSML)	*O*(*dD* ^3^ + *n* ^2^ *D*)
Kernel CSML	*O*(*dD* ^3^ + *n* ^2^ *D*)

**Table 2:** Classification performance comparison on the Yale database.

Method	2 Train		3 Train		4 Train		5 Train		6 Train		7 Train	
	Error (%)	*d *	Error (%)	*d *	Error (%)	*d *	Error (%)	*d *	Error (%)	*d *	Error (%)	*d *
PCA	56.6 ± 6.3	29	50.6 ± 8.3	44	47.4 ± 7.2	58	43.8 ± 6.5	74	40.8 ± 7.4	32	39.5 ± 5.1	30
RS-2DPCA	44.2 ± 6.0	17	32.5 ± 5.4	17	27.6 ± 5.9	17	22.4 ± 5.2	17	17.5 ± 6.3	17	16.1 ± 5.2	17
LDA	52.8 ± 7.5	10	35.1 ± 5.9	14	27.1 ± 5.3	14	21.2 ± 5.7	14	18.7 ± 4.9	14	17.6 ± 4.6	14
LPP	42.6 ± 6.8	14	31.2 ± 7.0	14	27.3 ± 6.2	19	21.1 ± 4.8	23	17.8 ± 5.8	24	16.3 ± 5.4	21
MFA	41.7 ± 7.4	18	33.6 ± 6.5	23	28.4 ± 5.9	27	21.5 ± 5.2	20	16.1 ± 5.3	19	15.2 ± 4.0	25
CDA	43.2 ± 5.9	19	32.9 ± 5.8	22	26.8 ± 6.7	23	20.3 ± 5.4	18	16.9 ± 6.8	26	16.0 ± 6.2	19
CEA	42.0 ± 6.1	21	30.7 ± 4.3	25	25.2 ± 4.9	18	19.2 ± 5.1	19	15.3 ± 5.4	20	14.1 ± 4.8	18
ISM-GE	43.1 ± 6.5	19	29.2 ± 5.9	20	23.6 ± 6.8	19	17.5 ± 6.3	20	14.7 ± 5.6	22	12.5 ± 5.1	19
MSE	42.4 ± 7.2	23	32.3 ± 6.7	24	28.2 ± 5.2	22	19.6 ± 5.9	21	16.2 ± 5.1	24	15.3 ± 5.7	22
CSML	**40.3** ± **6.8**	14	**29.4** ± **5.6**	19	**22.7** ± **6.1**	13	**17.8** ± **4.7**	20	**13.4** ± **4.2**	19	**11.7** ± **5.2**	15
KCSML	**37.4** ± **7.1**	18	**28.7** ± **6.3**	24	**23.1** ± **7.5**	21	**15.9** ± **6.0**	26	** 10.2** ± **5.4**	21	**9.6** ± **6.1**	27

**Table 3:** Classification performance comparison on the CMU PIE database.

Method	5 Train		10 Train		20 Train		30 Train	
	Error (%)	*d *	Error (%)	* d *	Error (%)	*d *	Error (%)	*d *
PCA	76.6 ± 4.3	334	64.8 ± 4.6	673	48.6 ± 3.8	982	37.9 ± 3.5	1023
RS-2DPCA	44.5 ± 4.1	18	28.3 ± 3.5	18	20.1 ± 2.6	18	9.6 ± 2.2	18
LDA	42.0 ± 3.6	67	29.7 ± 3.7	67	21.5 ± 2.9	67	10.9 ± 3.2	67
LPP	38.0 ± 4.8	67	29.6 ± 3.5	139	20.2 ± 3.3	147	10.8 ± 2.7	86
MFA	36.8 ± 4.4	72	28.2 ± 2.8	69	17.5 ± 2.6	68	9.8 ± 3.0	77
CDA	34.7 ± 3.9	85	23.5 ± 2.5	76	17.3 ± 2.3	79	8.9 ± 2.6	82
CEA	33.5 ± 4.2	241	22.1 ± 2.7	196	14.8 ± 1.9	283	8.4 ± 1.7	129
ISM-GE	32.6 ± 4.1	76	20.7 ± 3.2	73	11.3 ± 2.7	77	6.8 ± 1.6	79
MSE	34.9 ± 4.5	223	25.4 ± 2.5	226	19.0 ± 2.1	230	9.2 ± 1.9	221
CSML	** 30.4** ± **3.7**	201	** 16.8** ± **2.3**	215	** 9.2** ± **2.2**	194	** 6.1** ± **1.5**	192
KCSML	** 31.8** ± **4.3**	211	** 17.1** ± **2.9**	253	** 6.5** ± **2.4**	200	** 4.3** ± **2.1**	203

**Table 4:** Classification performance comparison on the MNIST database.

Method	50 Train		100 Train		150 Train		200 Train		250 Train		300 Train	
	Error (%)	*d *	Error (%)	*d *	Error (%)	*d *	Error (%)	*d *	Error (%)	*d *	Error (%)	*d *
PCA	16.1 ± 0.72	499	10.9 ± 0.46	517	9.2 ± 0.48	561	7.8 ± 0.33	578	7.0 ± 0.35	603	7.0 ± 0.28	610
RS-2DPCA	11.2 ± 0.42	18	7.3 ± 0.26	18	4.5 ± 0.21	18	3.8 ± 0.19	18	3.3 ± 0.20	18	2.0 ± 0.19	18
LDA	12.4 ± 0.53	9	9.2 ± 0.39	9	8.6 ± 0.27	9	7.0 ± 0.22	9	5.4 ± 0.24	9	4.6 ± 0.17	9
LPP	10.7 ± 0.47	56	6.7 ± 0.21	51	4.8 ± 0.25	43	3.5 ± 0.17	58	4.5 ± 0.14	69	1.9 ± 0.20	73
MFA	10.5 ± 0.42	114	7.1 ± 0.27	108	4.0 ± 0.23	121	3.7 ± 0.19	98	3.0 ± 0.17	105	1.8 ± 0.14	94
CDA	11.6 ± 0.38	49	6.2 ± 0.29	63	3.4 ± 0.26	70	3.3 ± 0.24	62	2.9 ± 0.22	54	2.2 ± 0.23	68
CEA	12.1 ± 0.43	31	5.9 ± 0.35	52	3.1 ± 0.16	62	3.8 ± 0.18	60	2.7 ± 0.17	83	1.6 ± 0.19	76
ISM-GE	10.8 ± 0.39	92	6.1 ± 0.32	91	3.3 ± 0.20	87	2.6 ± 0.17	94	1.9 ± 0.15	90	1.8 ± 0.17	91
MSE	11.5 ± 0.41	83	6.4 ± 0.28	85	3.7 ± 0.17	82	3.1 ± 0.22	87	2.7 ± 0.21	84	2.3 ± 0.19	83
CSML	** 9.4** ± **0.31**	62	** 5.3** ± **0.26**	79	** 3.6** ± **0.18**	85	** 1.9** ± **0.16**	83	** 1.3** ± **0.18**	83	** 1.2** ± **0.13**	89
KCSML	** 8.7** ± **0.36**	74	** 4.7** ± **0.34**	82	** 2.4** ± **0.16**	85	** 2.2** ± **0.17**	89	** 1.5** ± **0.12**	93	** 1.1** ± **0.16**	90
